# Supplementary material for: MYH11 rare variant augments aortic growth and induces cardiac hypertrophy and heart failure with pressure overload
Source: bioRxiv. 2024 Aug 16:2024.08.15.608063. Preprint. [Version 1] doi: 10.1101/2024.08.15.608063 (PMC11343208; doi:10.1101/2024.08.15.608063)
Supplement: Supplement 1 [file NIHPP2024.08.15.608063v1-supplement-1.pdf]

bioRxiv preprint doi: https://doi.org/10.1101/2024.08.15.608063; this version posted August 16, 2024. The copyright holder for this preprint (which was not certified by peer review) is the author/funder, who has granted bioRxiv a license to display the preprint in perpetuity. It is made available under aCC-BY 4.0 International license.

S1 Fig. Necropsy of a mouse died one day after transverse aortic constriction. One *Myh11*$^{E1892D/E1892D}$ female mouse died of ruptured left main coronary artery and associated cardiac tamponade one day after TAC. Yellow arrow and a 5-0 suture show the rupture site.

S2 Fig. Echocardiography and central blood pressure measurements 2 weeks after transverse aortic constriction (TAC) in female mice. **(A)** Aortic root and ascending (ASC) aortic diameters. **(B)** Systolic (SBP) and diastolic (DBP) blood pressures. **(C)** Evaluation of left ventricular (LV) contractile function in female mice after TAC. **(D)** Structural evaluation of LV in female mice after TAC. AW, anterior wall; PW, posterior wall; d, end diastolic; s, end systolic; ns, non-significant; * P<0.05.

S3 Fig. Wheat Germ Agglutinin (WGA) staining of left ventricular anterior wall (LVAW). There is no difference of cardiomyocyte cross-sectional area between male wild-type and *Myh11*$^{E1892D/E1892D}$ mice after TAC. ns, non-significant. ● WT TAC, ■ *Myh11*$^{E1892D/E1892D}$ TAC.

S4 Fig. Comparison of left ventricular contractile function between male and female mutant mice 2 weeks after transverse aortic constriction (TAC). Male *Myh11*$^{E1892D/E1892D}$ mice exhibit significantly lower ejection fraction and fractional shortening compared to female mutant mice following TAC. ns, non-significant; ** P<0.01.

S1 Table. Total numbers of mice died after transverse aortic constriction.

S2 Table. Segregation record of 102 progenies from heterozygous breeders.
